# Mechanisms Modified by (−)-Epicatechin and Taxifolin Relevant for the Treatment of Hypertension and Viral Infection: Knowledge from Preclinical Studies

**DOI:** 10.3390/antiox10030467

**Published:** 2021-03-16

**Authors:** Iveta Bernatova, Silvia Liskova

**Affiliations:** 1 Centre of Experimental Medicine, Institute of Normal and Pathological Physiology, Slovak Academy of Sciences, Sienkiewiczova 1, 813 71 Bratislava, Slovakia; silvia.liskova@savba.sk; 2 Faculty of Medicine, Institute of Pharmacology and Clinical Pharmacology, Comenius University, Sasinkova 4, 811 08 Bratislava, Slovakia

**Keywords:** dihydroquercetin, blood pressure, antioxidants, nitric oxide, Nrf2, antiviral, anti-inflammatory, red blood cell deformability, angiotensin II, COVID-19, SARS-CoV-2

## Abstract

Various studies have shown that certain flavonoids, flavonoid-containing plant extracts, and foods can improve human health. Experimental studies showed that flavonoids have the capacity to alter physiological processes as well as cellular and molecular mechanisms associated with their antioxidant properties. An important function of flavonoids was determined in the cardiovascular system, namely their capacity to lower blood pressure and to improve endothelial function. (−)-Epicatechin and taxifolin are two flavonoids with notable antihypertensive effects and multiple beneficial actions in the cardiovascular system, but they also possess antiviral effects, which may be of particular importance in the ongoing pandemic situation. Thus, this review is focused on the current knowledge of (−)-epicatechin as well as (+)-taxifolin and/or (−)-taxifolin-modified biological action and underlining molecular mechanisms determined in preclinical studies, which are relevant not only to the treatment of hypertension per se but may provide additional antiviral benefits that could be relevant to the treatment of hypertensive subjects with SARS-CoV-2 infection.

## 1. Introduction

The cardiovascular diseases (CVDs) (hypertension in particular) account for the most deaths of all noncommunicable diseases [1]. CVDs were also recognized as a risk factor that predisposes patients with severe acute respiratory syndrome coronavirus 2 (SARS-CoV-2)-infection to serious complications. Global age-standardized prevalence of raised blood pressure (BP) was 24.1% in men and 20.1% in women in 2015 [2], and it is rising with increasing age. In the ongoing coronavirus disease (COVID-19) pandemics, elderly people with pre-existing cardiovascular disorders in particular are vulnerable to developing SARS-CoV-2-induced complications that can be fatal [3,4].

**Figure 1:**
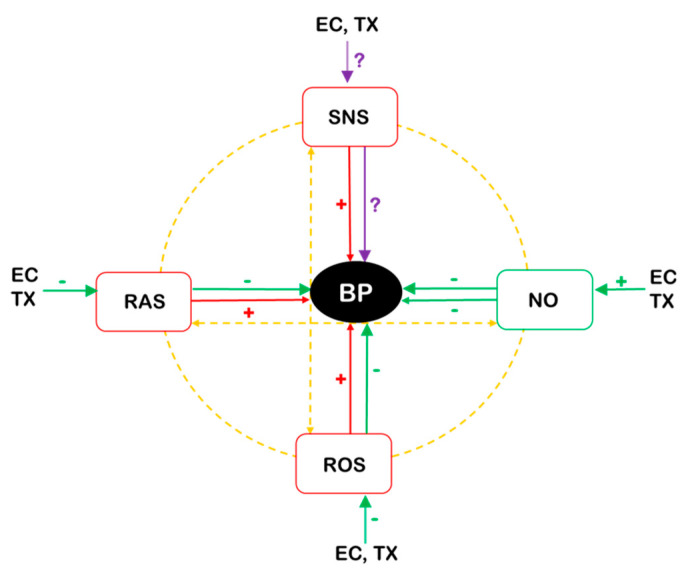
Scheme of interactions among the main blood pressure regulatory systems and reactive oxygen species. The SNS, RAS, ROS, and NO all participate in blood pressure regulation and interact with each other (orange lines). EC and TX were shown possess antioxidant properties and to reduce ROS. EC and TX also inhibit the RAS and elevate NO production (green lines). The SNS, RAS, and elevated ROS are known to elevate blood pressure (red lines). The effects of EC and TX directly on the SNS are unclear. Abbreviations: BP, blood pressure; EC, (−)-epicatechin; NO, nitric oxide; ROS, reactive oxygen species; SNS, sympathetic nervous system; RAS, renin–angiotensin system; TX, (+)-taxifolin, (−)-taxifolin or their racemic mixture.

The complex epidemiology and pathophysiology of hypertension development have been previously published in detail [5]. Briefly, BP is continually regulated by the central, peripheral, and local tissue regulatory mechanisms. Amongst the components of the BP regulatory network, the most powerful BP elevating systems are the renin–angiotensin system (RAS) and the sympathetic nervous system (SNS), and nitric oxide (NO) is the main depressor factor. All these mechanisms interact with each other and cooperate in the maintenance of optimal BP via central and peripheral vascular mechanisms (Figure 1). In addition, all of the BP regulatory systems interact with reactive oxygen species (ROS), which might lead to BP dysregulation and endothelial dysfunction, as oxidative stress has been observed in various experimental models of hypertension [6]. Moreover, membrane-bound angiotensin-converting enzyme 2 (ACE2), which is an intersection between the cardiovascular system (or blood pressure regulation) and viral infections [7], serves as the receptor of certain viruses. Importantly, Hoffmann et al. demonstrated that SARS-CoV-2 uses the ACE2 as the receptor to enter the cells [8].

Over the past two decades, various experimental and epidemiological studies have shown that the consumption of flavonoid-rich foods is associated with a reduced risk of CVDs [9,10]. Additionally, certain flavonoids were experimentally recognized as possible antiviral substances acting against viruses [11,12]. (−)-Epicatechin (EC) was found to inhibit the replication of hepatitis C virus [13] and Mayaro virus [14]. Green tea catechins and citrus flavonoids were identified as promising drug candidates for anti-COVID-19 treatment in the molecular docking studies [15,16]. (−)-Taxifolin was recognized as a possible inhibitor of SARS-CoV-2 replication by computational screening of a compound library of over 606 million of compounds [17] and by screening of a library of 44 citrus flavonoids [16].

This review is focused on the current knowledge on EC, and (+)-taxifolin and/or (−)-taxifolin (TX)-modified mechanisms found in preclinical studies, which may be relevant to the treatment of both hypertension and viral infection. These may provide synergic benefits in hypertensive subjects with SARS-CoV-2.

## 2. Flavonoids EC and TX

**Figure 2:**
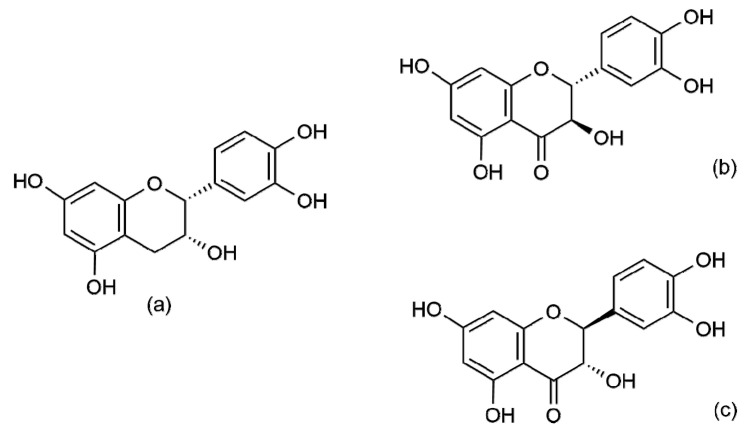
Structural formulas of (−)-epicatechin (**a**), (+)-taxifolin (**b**), and (−)-taxifolin (**c**).

(−)-Epicatechin (IUPACname(2*R*,3*R*)-2-(3,4-dihydroxyphenyl)-3,4-dihydro-2*H*-chromene-3,5,7-triol [18]) belongs to the subclass of flavan-3-ols (also known as catechins) together with (+)-epicatechin, (+)-catechin, and (−)-catechins [19]. Similarly, taxifolin has four stereoisomers: (+)-taxifolin (IUPAC name: (2*R*,3*R*)-2-(3,4-dihydroxyphenyl)-3,5,7-trihydroxy-2,3-dihydrochromen-4-one, also known as (2*R*,3*R*)-dihydroquercetin), (−)-taxifolin (IUPAC name: (2*S*,3*S*)-2-(3,4-dihydroxyphenyl)-3,5,7-trihydroxy-2,3-dihydrochromen-4-one), (−)-epitaxifolin (IUPAC name: (2*S*,3*R*)-2-(3,4-dihydroxyphenyl)-3,5,7-trihydroxy-2,3-dihydro-4H-chromen-4-one), and (+)-epitaxifolin (IUPAC name: (2*S*,3*R*)-2-(3,4-dihydroxyphenyl)-3,5,7-trihydroxy-2,3-dihydro-4*H*-chromen-4-one), which all belong to the subclass known as flavanonols [17,19,20] (Figure 2).

Both EC and TX are naturally present in various fruits, vegetables, and beverages. EC is present in beverages such as wine, beer, and juice but mainly in green tea, black tea, cacao, and cacao products (cocoa and chocolate). Cacao beans are supposedly the most abundant sources of EC [21,22].

TX can be found in red onions [23], apples [20], tomatoes, sorghum grain, white grapes, strawberries, mulberries [24], acai berries, peanuts, pine seeds [25], thyme, and citrus fruits, as well as beverages such as wine and beer [26]. TX is also present in a high concentration in conifers such as French Maritime Bark, Siberian Larch, Korean Red Pine, Cedar Deodar, Indian Pine and Chinese Yew, from which it can be industrially produced. Both EC and TX are also broadly available in various commercially available food supplements.

Flavonoids are consumed regularly in the human and animal diet; however, people’s daily intake of flavonoids is variable, depending on food resources in the given region, the season, and on the dietary habits of the specific population. Various studies showed a mean intake of flavonoids in the range of 190–450 mg/day [27,28,29]. The greatest daily dietary intake of flavonoids was estimated in the Kuna Indians, which was close to 900 mg EC. This was associated with low levels of BP even in the senior population [30].

## 3. Absorption and Toxicity of EC and TX

Regarding absorbability, EC can be absorbed better than other catechins in the gastrointestinal tracts of both rodents and people [31]. After ingestion, EC undergoes significant biotransformation and of its 67 metabolites detected in urine, nine (including EC itself) were found in circulation [32]. Four hours after administration, EC was also found in the heart and liver but not in the brain. EC is eliminated from an organism via renal and hepatic excretion [32].

Studies performed by Ottaviani et al. with radioactive EC investigating metabolism, distribution, and excretion in humans showed that about 82% of EC was quickly absorbed [33]. Radioactivity occurred in blood 15 min post EC ingestion. Its maximum in plasma was determined at about 60 min post ingestion. The concentration of EC metabolites determined at the same time in blood was about 1.22 μmol/L [33]. Several studies showed that flavanol metabolites in blood can cross the blood–brain barrier [34,35,36]. Importantly, EC was well tolerated in healthy people even after high daily consumption (2000 mg of cocoa flavanols per day for 12 weeks), without changes in the liver or blood metabolic parameters [37]. In rats, relatively high doses of EC (100 mg/kg/day, 2 weeks) did not produce significant signs of renal damage [38]. Furthermore, in a study conducted by [39], a harmful effect on the liver in mice treated with EC at a dose of 40 mg/kg/day for nine months was not observed.

TX, in contrast to many other flavonoids, is water soluble, its predicted water solubility is up to 1.16 g/L (Accession No. DB02224, www.drugbank.com), which is almost two times more than EC (0.645 g/L, Accession No. DB12039, www.drugbank.com, access date 30 January 2021). Better water solubility improves the bioavailability of TX in the organs and tissues in doses sufficient to induce biological effects [40]. In an earlier study conducted by Voskoboinikova [41], TX was found in blood at a concentration of no more than 0.5 pg/mL after single oral administration of TX to rats (500 mg/kg). TX was also detected in rats in plasma, and the kidneys, skeletal muscle, liver, heart, spleen, and brain [42]. In rabbits, single oral administration of a TX lipid solution led to a plasma TX concentration of about 590 ng/mL [43]. Studies also showed that TX, similar to EC, is a subject of considerable metabolism. Various metabolites of TX were previously found in humans and animals in the second half of the 20th century [44]. Recently, Yang et al. tentatively identified in total 191 TX metabolites, which resulted from TX hydroxylation, dehydroxylation, methylation, dehydrogenation, hydrogenation, dehydration, hydration, sulfation, and glucuronidation, respectively [45]. Of the these 191 metabolites, 127 were determined in urine, 83 in plasma, 43 in feces, and 46 in the organs (the heart, liver, spleen, lung, kidney, brain, stomach, and small intestine) of rats treated with 200 mg/kg/day TX for 3 days. Seventeen TX metabolites had bioactivities similar to those of TX alone [45].

A recent docking study conducted by Gogoi et al. assessed different toxicity parameters (mutagenic, tumorigenic, irritant, and reproductive effects) of TX and did not show any toxicity against the parameters used in the study, in contrast to quercetin, which showed the presence of mutagenic and tumorigenic properties [16]. Fischer et al. also reported low off-target (i.e., main protease M^pro^) binding properties of TX and, thus, its low predicted toxicity value [17]. Animal studies focused on TX-induced toxicity showed that TX is nontoxic even at high doses. In an earlier study, Booth and Deeds [44] showed that dietary administered TX to rats of both sexes for 226 days had no effect on body weight. Zhanateav et al. [46] showed that single administration of TX in doses of 15, 150, and 2000 mg/kg as well as repeated administration of TX in a dose of 0.15 and 1.5 mg/kg did not produce DNA damage in mouse bone marrow, blood cells, the liver, or the rectum. In addition, no significant chromosome aberrations in bone marrow cells of mice of both sexes were found. Oral TX administration to rats also failed to lead to behavioral alterations, lethalities, or organ damage [47]. Schauss et al. investigated the toxicity of TX-rich extract of a Dahurian Larch tree using variable dosing, routes of administration and toxicological tests (Comet assay, micronucleus test in human lymphocytes, chromosomal aberration test, subacute 7-day oral toxicity study, subchronic 90-day toxicology study with histopathologies, and prenatal and postnatal developmental toxicity studies) and provided evidence of the safety of TX [48].

Similar to EC, orally administered raw TX occurred in the plasma of rats at about 60 min post administration in a concentration of about 1.4 ng/mL [49]. Vega-Villa et al. investigated the stereospecific pharmacokinetics of TX stereoisomers and found that all four stereoisomers were present in serum 30 min after i.v. administration of racemic TX (40 mg/kg) and were also found in urine [20]. The same authors also showed that “stereoisomers of taxifolin are differentially distributed in the body. (+)-(2*S*;3*R*)-taxifolin is distributed intracellularly, (−)-(2*R*;3*S*)-taxifolin distributes extracellularly, while (−)-(2*S*;3*S*)- and (+)-(2*R*;3*R*)-taxifolin are deeply distributed into tissues. These findings suggest that the stereoisomers of taxifolin exit the vascular system and are either distributed intra- or extracellularly or penetrate deep into tissues depending on their stereochemical configuration” [20]. In humans, TX (free and conjugated) was detected in plasma up to 14 h after consumption of a single dose of 200 mg maritime pine bark extract, with the peak value ~40 ng/mL determined 8 h post administration. Interestingly, TX was not detected in plasma earlier than 2 h post oral administration [50].

On the other hand, nanoparticled TX was determined in the plasma of rats 9 min post gavage at a concentration of 13.5 ng/mL [49]. In addition, water-soluble nanostructures of TX (with cyclodextrins) prolonged an elevated concentration of TX in plasma by up to 7.5 h [51]. Improved water solubility of TX in the form of nanodispersion was also shown by [52]. There are also studies dedicated to investigating EC nanoparticles. Perez-Ruis showed an inhibitory effect of EC nanoparticles on human breast cancer cell lines, which was greater than the effect of free EC [53].

These data suggest that both EC and TX are well-tolerated, relatively nontoxic, and bioavailable flavonoids. In addition, novel methods of EC and TX preparation and treatment in the form of nanoparticles provide a promising way to increase the bioavailability of these flavonoids and their possible use as supplementary treatment in humans.

## 4. Biological Effects of EC and TX

As mentioned above, EC and TX are common components of the daily diet of humans and animals. In addition, they can also be found in the traditional medicine of various nations. Initially, in the modern research of flavonoids, a subgroup of flavonols was termed vitamin P [54]. Naturally occurring flavonoids with antiviral activity were first recognized in the 1940s. Currently, numerous studies show considerable health benefits of flavonoids which, among others, include cardiovascular, neuroprotective, and anticancer effects [55,56,57]. Concerning antiviral action against SARS-CoV-2, (−)-TX was recognized as a possible inhibitor of its main protease M^pro^ [17]. Importantly, (+)-TX was predicted to have about 26% less favorable binding free energies than (−)-TX [17], which also suggests that (+)-TX might have capability to act as an M^pro^ inhibitor, despite being less effective. Similarly, computationally guided identification of a citrus flavonoid found that TX is a potential inhibitor of SARS-CoV-2 of M^pro^ [16]. However, a docking study conducted by Ghosh et al. revealed that EC did not interact with the catalytic dyad of M^pro^ selected in their study [15].

In preclinical experimental research, the effects of EC and TX were extensively studied, and examples, although not exhaustive, of their biological activities that may be relevant to the treatment of hypertension and viral infection are listed in Table 1.

## 5. Cellular and Molecular Mechanisms of EC and TX Action Relevant to Cardiovascular Function

The mechanisms of EC and TX action in the cardiovascular system are mainly associated with antioxidant effects, alterations in NO production by endothelial (eNOS) and inducible NOS (iNOS), anti-inflammatory action, and modulation of the RAS and/or SNS. Another important role of EC and TX concerns the rheological properties of blood and deformability of red blood cells. Furthermore, the improvement in vascular function caused by EC and TX accounts for particularly important effects, as the prevention and treatment of endothelial dysfunction contribute to the improvement of blood flow and oxygenation of all tissues.

### 5.1. EC- and TX-Mediated Defense against Oxidative Stress

Currently, it is well known that ROS are involved in cellular homeostasis as the second messengers regulating various transcription factors and kinases. On the other hand, a disturbed redox balance towards oxidative stress is involved in the etiopathogenesis of cardiovascular and viral diseases [98,99]. In addition, oxidative stress seems to be the most prevalent cause of endothelial dysfunction in various experimental models of hypertension [6]. Thus, an optimal redox balance is essential for the maintenance of normal cell signaling and prevention of oxidative damage. A crucial role in redox balance plays the nuclear factor erythroid-2 related factor 2 (Nrf2)/Kelch-like ECH-associating protein 1 (Keap1) switch system [100,101,102]. Currently, approximately 250 genes are known to be regulated by Nrf2, and among them are various antioxidant genes and genes involved in iron metabolism and lipid metabolism [103].

The antioxidant action of flavonoids can be achieved through several mechanisms. Flavonoids can act as ROS scavengers, chelators of divalent metal cations (Fe^2+^, Cu^2+^, and Zn^2+^), or inhibitors of ROS-producing enzymes. Flavonoids can also terminate the chain reactions induced by ROS and interact with other initiators of chain reactions, thus attenuating further ROS production [104]. In addition, flavonoids alter iron metabolism and reduce the cytotoxic effects of Fe^2+^, namely ROS production in Fenton reaction.

EC and TX are potent scavengers for ROS. The scavenging action of EC in cell cultures was shown at concentrations as low as 0.5 µmol/L [105]. Other experimental evidence showed that EC and TX in the form of metal complexes possess superoxide scavenging capacity greater than that of the parent flavonoids. For example, the concentrations required to achieve 50% inhibition (IC50, determined under specific assay conditions) for the superoxide-driven reduction of nitroblue tetrazolium by EC and TX free ligands were 1.3 and 1.9 µmol/L, respectively. However, IC50s of their metal complexes were in range 0.3–0.6 µmol/L; lower (thus more effective) values were found for the EC–metal complexes than for the corresponding TX–metal complexes [106]. The experiments also demonstrated that these flavonoid–metal complexes are superoxide scavengers with superoxide-dismuting activity [106]. The chelation of Cu^2+^ ions by EC and TX, using the hematoxylin assay, was also confirmed by [107].

The ROS scavenging activities of TX and its dimer were investigated by Shubina et al. [108]. The authors found that the IC50s of TX and the TX dimer were 0.58 ± 0.04 µmol/L and 0.42 ± 0.03 µmol/L, respectively, using a luminol–H_2_O_2_–horseradish peroxidase system. The same study also confirmed the metal-chelating properties of TX and the TX dimer which created polyphenol-Fe^2+^ complexes in the stoichiometric ratio 1:2 and 1:4, respectively.

The antioxidant action of EC and TX is also associated with the activation of enzymes of the antioxidant defense system: superoxide dismutase (SOD), catalase (CAT), and glutathione peroxidase (GPx) [76,84]. The reduction of oxidative overload caused by flavonoids can be associated with changes in iron metabolism due to the reduction of iron absorption in the intestine. In the liver of iron-treated Wistar rats, TX reduced iron content and prevented lipid peroxidation, protein oxidation, and increased total antioxidant capacity via modulation redox-sensitive mitogen-activated protein kinase (MAPK) signaling [83].

Another antioxidant action of EC and TX is associated with the inhibition of nicotinamide adenine dinucleotide phosphate oxidase (NOX) and xanthine oxidase (XO) activities. IC50 values for the inhibition of NOX activity by EC and TX were >100 µmol/L and ~12 µmol/L, respectively. Lower IC50 were found for the inhibition of XO-dependent superoxide formation: ~5 µmol/L for EC and ~4 µmol/L for TX [109]. TX was more potent than quercetin in inhibiting superoxide produced by XO [96]. In mice, 3-day-long oral administration of TX in the dose of 100 mg/kg/day inhibited the activity of XO in the liver, leading to suppression of hepatic uric acid production [110]. This may also be an important protective mechanism since hyperuricemia was shown to contribute to the development of hypertension in humans [111], despite its failure in affecting vascular function of rats when determined ex vivo [112].

Importantly, the levels of EC and TX, in which antioxidant effects were experimentally determined, can be reached in the blood of humans or rodents after the consumption of EC, TX, or foods containing these flavonoids [33,42,113].

However, it is noteworthy that the levels of EC and TX in plasma and organs vary depending on the dose of these flavonoids administered, either as pure substances or as a component of plant extracts or food supplements (also containing other flavonoids), and on the microbiome, all of which may affect their absorption. In addition, flavonoids are able to also exhibit pro-oxidant activities depending on their concentration and on the metal (Fe^2+^, Zn^2+^, and Cu^2+^) concentrations [114].

As previously mentioned, Nrf2 serves as a transcription factor in the expression of various genes responding to oxidative load. Briefly, oxidative stress leads to the detachment of the Keap1 from its complex with Nrf2 [115]. Following the translocation of Nrf2 into the nucleus, Nrf2 interacts with the antioxidant response element (ARE) on the promoter of certain genes, and, thus, it facilitates the expression of ARE-containing genes [115]. Among many others, the group of the genes regulated by Nrf2 contains SOD, CAT, GPx, and NOX [115,116]. Another role of Nrf2 is the activation of certain genes, such as ferritin subunits L and H, transferrin, ferroportin, and hemoxygenase-1 (HO-1), involved in iron metabolism, among which ferritin and HO-1 participate in antioxidant defense [117,118]. Nrf2 can be thus considered a key factor of redox regulation in CVDs [119], as it regulates cellular responses to oxidative stress and inflammation together with the nuclear factor-κB (NF-κB) [120].

EC and TX were shown to induce Nrf2 expression in vitro [121]. In in vivo experiments, EC prevented stroke damage through the activation of Nrf2 and increase in HO-1 in mice [72]. In Wistar rats, EC (10 mg/kg) increased the transcription of the Nrf2 gene and Nrf2 target genes in the aorta [61].

Less information is available regarding Nrf2 activation by TX. TX pretreatment prevented ischemia–reperfusion-induced myocardial injury, alleviated cardiac dysfunction, scavenged ROS, reduced lipid peroxidation, and increased the activity of antioxidant enzymes (CAT, SOD, and GPx) in isolated rat hearts. In addition, TX reduced proapoptotic protein expression in association with increasing protein expressions of HO-1 and Nrf2 [76]. Similarly, TX was shown to activate the Nrf2 antioxidative stress pathway via increases in Nrf2-ARE activity by upregulating HO-1 and NAD(P)H quinone oxidoreductase 1 expression in mouse skin epidermal cells through epigenetic modifications [40]. The protective mechanism of TX via the activation of Nrf2/HO-1 pathway was confirmed even during organophosphorus pesticide damage of microglial cells [122]. In addition, significant analysis of the microarray in human colon carcinoma cell cultures revealed 65 genes, including detoxification enzymes (NAD(P)H quinone oxidoreductase 1, glutathione S-transferase M1, and an antioxidant enzyme (thioredoxin reductase 1), which were upregulated, while another 363 genes were downregulated in the presence of TX (60 mmol/L), suggesting a strong potential of TX to act as a chemopreventive and/or antioxidant agent [123].

### 5.2. NO-Mediated Effects of EC and TX

**Figure 3:**
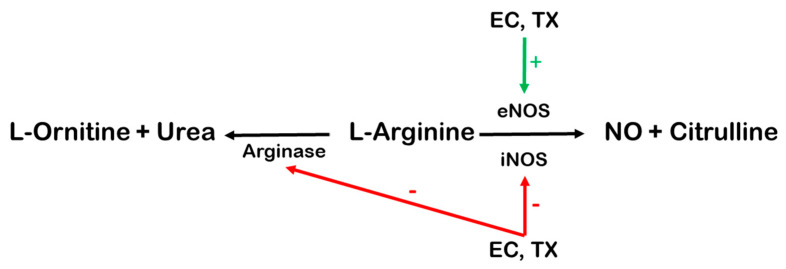
The EC- and TX-induced beneficial mechanisms involved in NO production. The green arrow indicates stimulation of the enzyme, which leads to the augmentation of NO production by eNOS. The red arrows indicate the EC- and TX-induced inhibition of the enzymes, leading to downregulation of iNOS in injured tissue and the inhibition of arginase. Abbreviations: EC, (−)-epicatechin; eNOS, endothelial nitric oxide synthase; NO, nitric oxide; TX, (+)-taxifolin, (−)-taxifolin or their racemic mixture.

Several studies showed that the beneficial effects of flavonoids could be attributed to the increase in NOS activity in vasculature, mainly endothelial NOS (eNOS, Figure 3). In human coronary artery endothelial cells, EC-induced eNOS activation occurs in the presence of Ca^2+^ via the phosphatidylinositol-3-kinase/protein kinase B/protein kinase A (PI3K/Akt/PKA) and Ca^2+^/calmodulin-dependent kinase II [124]. Moreno-Ulloa et al. demonstrated that in Ca^2+^-free medium, EC also induced eNOS activation via the phosphorylation of PI3K/Akt, which appeared to be coupled to a cell membrane receptor [125]. In another study, EC stimulated NO production via the formation of an active complex between eNOS, Akt, and heat shock protein 90 [126]. Furthermore, EC administered at a dose of 1 mg/kg for 15 days restored both eNOS and plasma nitrite/nitrate levels in mice with high-fat-diet-induced eNOS dysfunction in the myocardium [127].

On the other hand, the increased activity of inducible NOS (iNOS) isoform is mostly involved in pathological conditions, and, thus, the beneficial effect of EC and TX may be associated with the inhibition of iNOS.

Upregulation of iNOS was shown, for example, in the lung fibroblasts in association with the early proliferative response to cytokine stimulation [128]. In the model of bleomycin-induced lung injury, increased protein expression of iNOS was reduced after TX administration (10 mg/kg/day for 7 days) [129]. TX administration resulted in the downregulation of iNOS in concanavalin A-induced immunological hepatic injury of mice [130]. Similar inhibition of iNOS expression was reported during lipopolysaccharide-induced renal damage in Sprague–Dawley rats fed a diet containing EC (80 mg/kg body weight/day) for 4 days [87]. In the cell culture of human keratinocyte (HaCaT), the lipopolysaccharide-induced expression of the iNOS protein decreased almost to the control value when the HaCaT cells were preincubated with TX (100 µM/24 h) and decreased to about 50% after EC (100 µM/24 h) preincubation [131]. Thus, only TX preincubation significantly inhibited NO production, presumably via the downregulation of iNOS [131]. Prolonged incubation of HaCaT cells with EC may also lead to the inhibition of NO production via inhibition of iNOS, because in another study, EC treatment was able to decrease serum NO levels to the control value in mice fed D-galactose [90]. A similar decrease in serum NO levels was observed after TX treatment [90]. These results point to a reduction of iNOS activity after EC or TX application.

In addition, because arginase competes with NOS for the substrate L-arginine, the beneficial effect of EC and TX may be further strengthened by the inhibitory action of EC and TX on the arginase activity (Figure 3). The bovine coronary artery endothelial cells incubated with EC (1 μM) for 48 h showed decreased arginase activity and increased NO generation [58]. Arginase 1 and arginase 2 protein levels were increased by ischemia–reperfusion in mice hearts, and EC treatment (1 mg/kg/day for 10 days) attenuated these increases [132]. EC and TX also inhibited mammal arginase activity with IC50 ~20 µM [133]. Altogether, both EC and TX can inhibit arginase activity and, thus, improve substrate availability for NO production.

The abovementioned studies provide solid evidence that EC and TX applications increase the eNOS-derived NO production leading to the improvement of cardiovascular function and decreased inflammatory response through the reduction of iNOS.

### 5.3. Anti-Inflammatory Effects of EC and TX

In addition to Nrf2-mediated antioxidant effects, the functional cross-talk between Nrf2 and NF-κB represents a switch between oxidative stress and inflammation [120]. During inflammation, the production of cyclooxygenase 2 (COX-2), tumor necrosis factor α (TNFα), iNOS, and proinflammatory interleukins (IL-1β, IL-6, IL-12, and IL-18) is elevated within the cell. The absence of Nrf2 in mouse primary cultured astrocytes exacerbated NF-κB activity, leading to increased cytokine production [134]. In contrast, under oxidative stress, Nfr2-induced transcriptional activation of HO-1 can lead to the inhibition of NF-κB, resulting in a decrease in proinflammatory cytokine levels [135].

Inflammation, which participates even in hypertension development [136], was shown to be reduced by EC in the vasculature via the inhibition of NFκB [137]. EC decreased the release of proinflammatory factors such as TNF-α and the monocyte chemoattractant protein-1 from adipocytes [138]. EC also reduced acute intestinal inflammation [86]. Similarly, EC prevented the renal damage induced by lipopolysaccharide administration in Sprague–Dawley rats by the reduction of iNOS (Figure 3), IL-6, TNFα, and NF-κB p65 subunit expression [87]. Recently, Kang et al. showed that EC (administered as a supplement of 20 mg EC/kg in the diet) attenuated plasma endotoxin levels and parameters of neuroinflammation in the hippocampus (mRNA levels of Toll-like receptor 4, ionized calcium binding adaptor molecule 1, and NOX4) of high-fat diet-fed C57BL/6J mice [88].

Anti-inflammatory effects of TX were previously shown in an experimental model of arthritis in rats in 1971 [139]. In the liver of iron-treated Wistar rats, TX reduced iron content and proinflammatory cytokines TNF-α, IL-1β, and IL-6 [83]. TX also ameliorated cerebral ischemia–reperfusion injury in rats through its antioxidative effect and modulation of NF-κB activation [140]. In the same study, the authors showed that TX inhibited leukocyte infiltration as well as COX-2 and iNOS expressions in an injured brain [140]. Importantly, TX also showed kidney protection effects by suppressing nucleotide binding and the oligomerization domain-like receptor family pyrin domain-containing 3 (NLRP3) inflammasome [141]. TX also protected the rat ventricular H9C2 cells from oxidative injury and suppressed H_2_O_2_-induced cell pyroptosis via the inhibition of both NLRP3 and nucleotide-binding oligomerization domain-like receptor family caspase recruitment domain-containing protein 4 (NLRC4) inflammasomes [142]. It is important to note that activated NLRP3 triggers an immune response [141], which is activated in response to SARS-CoV-2 infection and is active in COVID-19. The inflammasome has been suggested as “a marker of disease severity and a potential therapeutic target for COVID-19” [143].

The abovementioned studies support the idea that the consumption of EC and TX may reduce inflammation and/or cytokine storm in subjects infected with SARS-CoV-2 virus, thereby providing additional benefits of these flavonoids in the treatment of COVID-19.

### 5.4. EC and TX-Mediated Effects on the SNS and the RAS

The interactions between the SNS and RAS play an important role in hypertension development even when hypertension is induced by distinct pathophysiological stimuli in animal models [144,145,146]. The enhanced SNS activity, stimulation of the RAS, increased release of noradrenaline and adrenaline into circulation, and renal vasoconstriction are known hallmarks of hypertension.

Recent studies showed that the direct effects of flavonoids or polyphenol-rich foods on the autonomic nervous system and the central RAS are unclear, and variable effects have been observed [147]. Studies on EC showed that it failed to change the plasma adrenaline levels in a murine study when administered at a single dose of 10 mg/kg [148]. Similarly, 1 mg/kg EC failed to alter plasma noradrenaline and adrenaline levels 2 h postingestion in mice [149]. These results were obtained in normotensive mice, but EC may be able to lower the plasma catecholamines level when it is elevated, as, for example, in hypertension, because a stress-induced increase in catecholamines was prevented after ingestion of dark chocolate in a clinical trial [150].

It is well known that there is cross-talk between the SNS and RAS [151]; the activation of the SNS tone activates the RAS [152]. On the other hand, RAS exerts several actions on the SNS, such as a central action to increase sympathetic outflow, stimulatory effects on the sympathetic ganglia and the adrenal medulla, and actions on sympathetic nerve endings [153].

**Figure 4:**
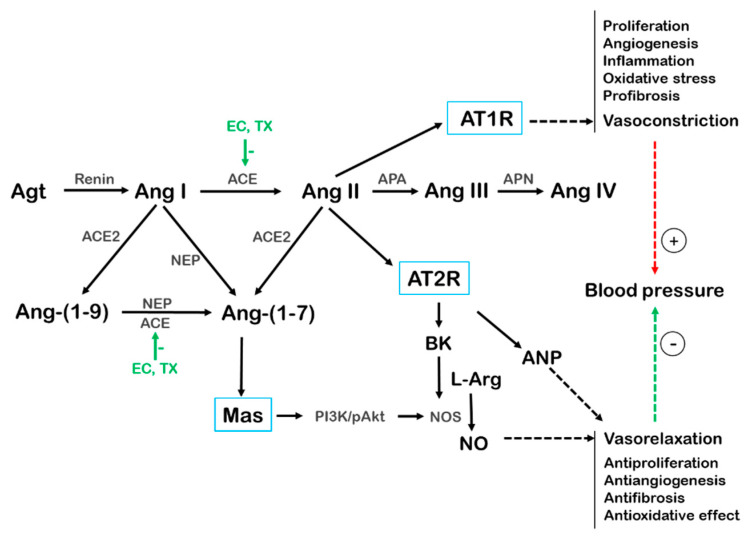
A brief, although not exhaustive, scheme of the renin–angiotensin system. Abbreviations: ACE, angiotensin converting enzyme; Agt, angiotensinogen; Ang, angiotensin; ANP, atrial natriuretic peptide; APA, aminopeptidase A; APN, aminopeptidase N; AT1R, angiotensin II type 1 receptor; AT2R, angiotensin II type 2 receptor; BK, bradykinin; EC, (−)-epicatechin; L-Arg, L-arginine; Mas, Mas receptor; NEP, neutral endopeptidase, NO, nitric oxide; NOS, nitric oxide synthase; PI3/Akt, phosphatidylinositol 3-kinase/kinase B; TX, (+)-taxifolin, (−)-taxifolin or their racemic mixture.

The main effector of the RAS is angiotensin II (Ang II). Briefly, the first enzyme in the RAS cascade is renin, which is secreted by the kidneys and cleaves angiotensinogen to Ang I (Figure 4). This is cleaved by ACE to Ang II [154]. Both Ang I and Ang II are further cleaved by ACE2 to Ang-(1–9) and Ang-(1–7), respectively. Ang-(1–9) is converted to Ang-(1–7) by ACE and neutral endopeptidase (NEP). The cleavage of Ang II to Ang III and Ang IV by aminopeptidases A and N, respectively, is also known. Several angiotensin receptors were described, namely AT1R (AT1a, AT1b), AT2R, AT3R, AT4R, and Mas, which mediate their downstream signaling. AT2R is known to oppose the effects of AT1R, but there are relatively few data on the effects of AT3R and AT4R [144]. Ang-(1–7) is a vasodilator; it decreases BP, reduces vascular cell growth, and preserves both cardiac and aortic endothelial functions [154]. It mainly acts via the G-protein-coupled Mas receptor. Mas signaling is associated with PI3K/Akt/PKA activation and NO production [155]. The distribution of angiotensin receptors in tissues varies, but the machinery of Ang II production has been found in the brain, vascular endothelium, heart, kidneys, adipose tissue, and other organs and tissues [154]. In addition, the RAS is present in the lungs, and is important in the pathogenesis of acute respiratory distress syndrome; additionally, the reduction of ACE2 can participate in the development of pulmonary fibrosis, as reviewed in detail by Hrenak and Simko [156].

Importantly, it has been shown that the membrane isoform of ACE2 serves as a receptor for certain coronaviruses, including SARS-CoV-2 [157], via which this virus enters the cells. The binding of the virus to membrane ACE2 results in the dysfunction of Mas receptor-mediated pathways in the vasculature, reduces downstream NO release, and promotes endothelial dysfunction [157,158]. Moreover, in the endothelium, Ang II acts as one of the endothelium-derived contracting factors (EDCFs) as described below. In hypertension, inhibition of ACE and/or elevation of ACE2 activities reduce BP. On the other hand, during SARS-CoV-2 infection, loss of normal ACE2 function may worsen existing CVDs [157]. These physiological roles of ACE2 also indicate that the reduction of ACE2 signaling either by ACE2 inhibitors or by flavonoids might not be a suitable way to block SARS-CoV-2 entry into the cells.

Flavonoids can also alter the RAS. Even though the study conducted by Li et al. showed that EC failed to inhibit renin activity [159], the inhibitory effect of EC and other tea catechins on ACE was shown in an in vitro study by [160]. Next, the dose-dependent inhibition of ACE activity was also observed in cultured endothelial cells from human umbilical veins (HUVEC), when they were incubated with EC [161]. Montes-Rivera et al. showed that EC reduced BP and AT1R expression in the model of 5/6 nephrectomy in mice when EC was administered at the low dose 1 mg/kg/day for 14 days [162]. A review of the literature conducted by Muchtaridi et al. showed that various flavonoids have the potential to inhibit ACE2 [163]. There are also studies that showed the effects of EC and TX, respectively, on the RAS or ACE in vivo in conditions of experimental hypertension, which are mentioned below.

An important role in the ACE-inhibitory mechanism of EC play hydroxyl groups at the B-ring, the covalent modification of ACE by tea catechins [160], and the chelation of Zn^2+^ ion at the active center of ACE [164]. In addition, Guerrero et al. provided a structure–activity relationship study which, among others, showed that the loss of the 3-OH, 3′-OH, and 5′-OH groups significantly reduced the ACE inhibitory activity of various flavonoids, including EC [165].

### 5.5. Mechanisms Involved in the Vascular Action of EC and TX

The endothelium produces endothelium-derived relaxing factors (EDRFs, mainly NO and prostacyclin) and EDCFs (mainly prostaglandins, endothelin-1, Ang II, and ROS). Dysregulation of the cross-talk between EDRFs and EDCFs results in the alteration of normal physiological processes carried out by the endothelium, including reduction of its anticoagulant and antithrombotic properties, acceleration of vascular growth and remodeling, and impairment of endothelium-dependent vasorelaxation, i.e., in endothelial dysfunction [6]. Endothelial dysfunction was documented not only in animal models, but also in patients with arterial hypertension [166]. Prevention of endothelial dysfunction represents an important step in the prevention or mitigation of CVDs and hypertension. Moreover, Libby and Luscher have recently provided the concept of COVID-19 as an endothelial disease, which provides a unifying pathophysiological picture of SARS-CoV-2 infection [167].

Using isolated aortic rings with an intact endothelium from Sprague–Dawley rats, direct EC application into the organ chamber caused relaxation, whereas endothelium removal or NOS inhibition abolished EC-induced relaxations [67]. In different study on Wistar rats, after NOS inhibition, EC actually induced contraction at resting tension (without precontraction) of the aortic rings [68], showing the necessity of the endothelium in the beneficial effect of EC. When thoracic aortic rings were precontracted with noradrenaline, EC-induced relaxations were mediated through the involvement of voltage-dependent calcium channels and an increase in NO release from the endothelium [68]. Furthermore, in aged rats (18 months old) EC (1 mg/kg/day for 15 days) improved acetylcholine-induced relaxation of the aortic rings [58]. The improvement in endothelium-dependent relaxation after EC administration was associated with elevated eNOS expression in aortic rings of young spontaneously hypertensive rats (SHRs) [38], while elevated phosphorylated eNOS protein levels were found in deoxycorticosterone acetate (DOCA)/salt hypertensive rats [61].

In the femoral artery, 10-day dietary administration of EC to adult SHRs prevented blood pressure increase and augmented acetylcholine-induced endothelium-dependent relaxation via elevation of its NO-dependent component [59].

In mesenteric arteries precontracted with KCl, EC induced relaxation by induction of NO release via voltage-dependent calcium channels and voltage-dependent potassium channels; in contrast, after noradrenaline precontraction, EC induced mesenteric artery contraction after the inhibition of voltage-dependent potassium channels [68]. EC reduced phenylephrine-induced contractions in the mesenteric arteries isolated from Sprague–Dawley rats [168] and induced endothelium-dependent relaxation mediated through NO [168,169].

EC also induced concentration-dependent relaxation of human internal mammary arteries and human saphenous veins through the voltage-gated K^+^ channels (mainly KV1.3 channels), ATP-sensitive K^+^ channels, and large conductance Ca^2+^-activated K^+^ channels, as well as voltage-dependent Ca^2+^ channels [69,70].

In addition, Ramirez-Sanchez et al. showed that EC stimulated myocardial angiogenesis in 1-year-old mice, and this was associated with increases in protein levels and/or the activation of events associated with the canonical angiogenesis pathway (i.e., with the elevation/activation of hypoxia inducible factor 1a, vascular endothelial growth factor, vascular endothelial growth factor-R2, PI3K, pyruvate dehydrogenase kinase, Akt, eNOS, NO, cGMP, matrix metalloproteinase-2/-9, steroid receptor coactivator-1, and a cluster of differentiation 31 protein) [170]. Thus, in addition to the improved endothelial function, EC may represent a way to stimulate angiogenesis [170].

However, there are still questions associated with the vascular receptor activated by EC. Chalopin et al. suggested that estrogen receptor α may mediate the vascular effects of certain natural polyphenols [171]. Indeed, Moreno-Ulloa et al. found that EC induced an increase in NO production by eNOS in endothelial cells via the G protein-coupled estrogen receptor [172]. In addition, MacRae et al. showed that EC vascular responses and cardioprotective effects were mediated through the opioid receptors [68]. The same authors showed that further mechanisms of EC action in the vasculature involve modulation of potassium and calcium channels, which participate in the hyperpolarization and depolarization of vascular smooth muscle cells [68]. Recent studies of Ortiz-Flores et al. have shown that EC interacts with and activates the pregnane X receptor [173]. There is a limited number of studies that investigated the vascular effects of TX. In vitro administration of TX (into the organ chamber) caused concentration-dependent relaxation of phenylephrine-precontrated thoracic aorta rings isolated from SHRs [64]. Despite the fact that the exact vascular receptor of TX is not known, the improvement in vascular function may depend on the inhibition of signaling pathways leading to vascular dysfunction. Very recently, Cao et al. showed that TX could reverse the effects of Cr(VI)-induced cell damage in human umbilical vein endothelial cells by inhibiting the activation of mitogen-activated protein kinases and NF-κB signaling pathways, demonstrating that TX could prevent endothelial dysfunction [174].

The abovementioned studies showed that the vascular effects of EC and TX vary depending on artery type, dose, or animal species used, as well as on the duration of treatment. We note that the vascular effects of EC and TX were also investigated in conditions of experimental hypertension, and these are mentioned below.

### 5.6. EC and TX Effects on Blood Cells and Rheology

It is well known that the rheological properties of blood are directly involved in the regulation of blood pressure and hypertension development [175,176].

In experimental models, the deformability of red blood cells was reduced in NO-deficient hypertension (NODH), while red blood cell aggregation was elevated in NODH, DOCA, and the two kidney–one clip model of hypertension [177]. Similarly, reduced erythrocyte membrane deformability was found in 3-week-old SHRs [178], while dose-dependent fibrinogen-induced red blood cell aggregation was found in adult rats [179]. Furthermore, it was found that primary hypertension is associated with an increased risk of arterial thrombotic disease, associated with by endothelial dysfunction, decreased platelet NO biosynthesis, and platelet degranulation, secondary to increased shear stress [180].

In the plasma of healthy subjects, platelet microparticles and thrombin generation formation (upon platelet activation) were reduced with increased concentrations of EC (1 to 100 μM, in vitro), leading to reduced procoagulant properties [93]. In addition, a reduction of maximal platelet aggregation induced by adenosine diphosphate (ADP) and decreased generation of the thrombin-receptor-activating peptide after EC administration (100 μM) to plasma samples in vitro were reported [92]. Thus, EC reduced endogenous thrombin potential and promoted fibrinolysis, suggesting that decreased platelet function results in an anticoagulant and profibrinolytic profile, which can be beneficial in CVD prevention [92].

Similarly, TX inhibits platelet aggregation induced by ADP and thrombin release [95]. Kubatiev et al. showed that TX causes a concentration-dependent decrease in cytoplasmic Ca^2+^ levels in thrombocytes induced by ADP or thrombin probably via the Ca^2+^ inhibition channels [94]. Higher concentrations of TX decreased the basal cytoplasmatic Ca^2+^ concentration probably due to the elevation in the cAMP level. Similar inhibition of ADP-induced thrombocyte aggregation by TX was reported by [96]. Furthermore, TX provided protection against hemolysis induced by free radicals as well as against nonoxidative hemolytic enzyme phospholipase C [96]. A complex of TX and ascorbic acid decreased the aggregation, increased the deformability of erythrocytes, and reduced lipid peroxidation in erythrocytes membrane and blood plasma of aged patients [91].

These studies showed that both EC and TX may provide protection against red blood cell aggregation, coagulation, and thrombogenesis and that they can elevate red blood cell deformability, which can be another beneficial effect for subjects suffering from hypertension and/or COVID-19.

Cellular and molecular mechanisms underlying the biological effects of EC and TX are listed in the Table 2.

## 6. Mechanisms of EC and TX Action in Experimental Models of Hypertension

Antihypertensive and vascular effects of EC and TX were investigated in several experimental models of hypertension, which differ in the primary mechanism of BP elevation. In this review, we focus on a model of SHRs, borderline hypertensive rats (BHRs), and NO-deficient (i.e., N^G^-nitro-L-arginine methyl ester (L-NAME)-induced) model of hypertension, and selected metabolic models such as DOCA/salt-induced hypertension and high fructose-induced hypertension.

### 6.1. EC and TX Action in Spontaneously Hypertensive and Borderline Hypertensive Rats

The most frequently used experimental model of human arterial hypertension is the SHR. However, in SHRs, hypertension (comparable with stage-two human arterial hypertension) and endothelial dysfunction develop in early life, usually between the 7th and 12th week of age, i.e., in young rats, which is in contrast to humans, where hypertension develops mainly in the later period of life [189,190]. Thus, BHRs are a suitable experimental model to study the early stages of hypertension (prehypertension) in late adulthood [191,192].

In our studies, we investigated the cardiovascular effects of EC in adult SHRs as well as in young SHRs and BHRs. In adult SHRs, EC significantly reduced BP after 2 days of treatment when a relatively high dose of EC was used (~250 mg/kg/day). This was associated with the finding that elevated vascular NOS activity improved endothelium-dependent acetylcholine-induced relaxation in the femoral artery, namely with its NO-dependent component, which was even greater than that in age-matched Wistra–Kyoto rats [59]. In contrast, EC administered to adult SHRs at the low dose (5 mg/kg/day) for 20 weeks failed to reduce BP, despite the fact that plasma NO was elevated and markers of oxidative stress were reduced [193].

In young, 7-week-old SHRs treated with EC (~100 mg/kg/day) for 2 weeks, a significant attenuation of age-dependent BP increase was found in line with increased NOS activity and reduced superoxide production in the aorta and the left heart ventricle. EC also increased the total antioxidant capacity of plasma and the erythrocyte deformability [38]. The same EC treatment regimen in young BHRs led to reductions of BP, which persisted for the following two weeks after cessation of EC treatment [60]. EC also reduced superoxide production in the aorta but not in the left heart ventricle. Elevated NOS activity was found in both the left heart ventricle and aorta despite the fact that the expressions of Nrf2 and eNOS/iNOS/neuronal NOS genes were unaltered [60]. In addition, chronic EC treatment reduced iron levels in the blood of young BHRs [60].

The effects of TX in the experimental hypertension model are less studied compared to EC. Treatment of SHRs with TX (20 mg/kg/day intragastrically) for 6 weeks reduced BP by 11% and whole blood viscosity by 7–10% vs. control SHRs. In addition, half-time erythrocyte aggregation of TX-treated rats increased, while erythrocyte deformability did not change [64]. The same study also showed vasorelaxing effects of TX when using an isolated aorta. On the other hand, a 2-week-long treatment of SHRs with TX (300 µg/kg) failed to reduce BP and ACE activity in the aorta, while the same dose of TX reduced ACE activity in WKY rats [186]. In contrast to EC, TX did not affect the age-dependent development of hypertension in SHRs, but it improved microcirculation of the brain tissue when administered at a dose of 50 mg/kg/day for 6 weeks intragastrically [71].

A recent study conducted by Kim et al. investigated the influence of the polyphenolic extract from Korean Red Pine Bark, which contains TX (together with protocatechuic acid, procyanidin B1, catechin, caffeic acid, and vanillin) [65]. This extract reduced BP of SHRs to a similar level as ACE inhibitor captopril following a 7-week treatment period. TX also reduced ACE activity in serum and the lungs, and Ang II content and lipid peroxidation in serum, the lungs, and the kidney to levels comparable with those of the captopril-treated group [65].

These studies indicated the potential of EC and TX to reduce BP and improve vascular function in rats with spontaneously elevated BP, which was associated mainly with their antioxidant effects, elevation of NOS activity, and reduction of ACE activity.

### 6.2. EC and TX Action in NO-Deficient Hypertension

Another frequently used rat model of hypertension is the model of NODH induced by chronic administration of the nonspecific NOS inhibitor L-NAME. In this model, the increase in BP is induced by the inhibition of NO production. NO-deficient hypertension is associated with reduced endothelium-dependent relaxation and increased contraction in different arteries [194,195,196]. NOS inhibition also induces endothelium-dependent contractions by elevated production of EDCFs and ET-1 [197,198]. In addition to the altered vascular function, NODH also involves the elevated tone of the SNS, accentuation of the RAS, and the induction of oxidative stress [147,199,200]. Lack of NO also results in vascular remodeling [201].

Regarding EC effects in NODH, the L-NAME-induced increase in BP was reduced during simultaneous administration of EC (approximately 300 mg/kg/day) and L-NAME for 4 days; however, cessation of EC treatment led to BP increase. EC treatment also prevented the decrease in NOS activity when administered simultaneously with L-NAME. EC also buffered an increase in superoxide production and NOX p47phox subunit protein expression [184]. Similarly, 4-day simultaneous EC administration (4 g/kg of food) in L-NAME-treated Sprague–Dawley rats prevented the BP increase, because EC administration restored NOS activity via phosphorylation of eNOS and reduced superoxide production due to the decrease in the p47phox subunit of NOX [63]. However, in another study, a lower dose of EC (10 mg/kg/day) failed to prevent the increase in BP despite the fact that it reduced vasoconstriction, increased eNOS and Akt phosphorylation, prevented the L-NAME-induced oxidative stress, and reduced proinflammatory markers [181]. Recently, Prince et al. investigated the effect of EC on kidneys in L-NAME-treated rats [182]. The authors found that EC administration restored kidney function parameters; oxidative stress markers; expression of p47phox, gp91phox, and NOX4; and NOS activity to the values of the control group. EC also restored activities and/or expressions of antioxidant enzymes such as Mn-SOD, GPx, and CAT, but not CuZn-SOD [182].

To our knowledge, there is only one available study that investigated the effect of TX in NODH. In that study, L-NAME (100 mg/kg/day) and TX (10, 30, or 100 μg/kg/day) were administered in drinking water. TX treatment at a doses 30 and 100 μg/kg/day, respectively, blocked the elevation of ACE activity, which remained similar to that observed in the control group. In addition, administration of TX at a dose of 100 μg/kg/day for 5 days reduced ROS/reactive nitrogen species formation in the aorta [187].

### 6.3. Mechanisms of EC and TX Action in Other Models of Hypertension

One of the most commonly used rat models of hypertension is the DOCA/high-salt diet model, which is considered a neurogenic model of hypertension, but it also has a significant renal, cardiovascular, and immune component [202]. In DOCA/salt-induced hypertension, chronic EC administration (10 mg/kg/day) reduced BP. In the aorta, EC reduced aortic superoxide release in line with reduced NOX activity, plasma endothelin-1, and urinary 8-iso-prostaglandin F_2α_ excretion. EC also improved endothelium-dependent relaxation and increased the phosphorylation of both Akt and eNOS in the aortic rings [61]. Moreover, EC treatment (10 mg/kg/day, 5 weeks) increased Nrf2 protein expression in the nuclear fraction of aortic rings together with the decrease in NOX activity and improvement of vasorelaxation [61].

In the same model of hypertension, French Maritime Pine Bark extract, which consisted of catechin, TX, and proanthocyanidins, had an antihypertensive effect [66]. This was associated with reduced superoxide production, protective effects against endothelial dysfunction in the isolated aortic rings, and improvement in relaxation in the mesenteric bed. This extract also induced an increase in total eNOS and phosphorylated eNOS expression in the aorta. However, this feeding of rats failed to suppress the development of hypertension in the presence of L-NAME [66], suggesting that NO is a mediator of the protective effects of TX.

Similar findings exist for hypertension induced by a high-fructose diet. The mechanism of high-fructose-induced hypertension is not fully understood; however, high-fructose intake is associated with upregulation of sodium and chloride transporters resulting in a salt overload that increases BP probably via endothelial dysfunction and stimulation of the SNS [203]. Indeed, the high-fructose diet reduced NO levels in the nucleus tractus solitarii and the consequent baroreflex dysfunction further stimulated SNS activity, leading to the development of high BP [204] and impairment of endothelium-dependent relaxation in the isolated aortic rings [205], without alterations in contractions of isolated superior mesenteric arteries [206]. In this model, dietary EC administered at a dose of 20 mg/kg/day prevented BP increase and reduced superoxide anion production and the expression of the NOX subunits in the aorta; it also accentuated NO production and expression of phosphorylated eNOS. In addition, EC supplementation mitigated high fructose-mediated c-Jun N-terminal kinase activation in the aorta, but it did not affect its structure [62]. In another study, Calabro et al. showed that 8-week EC supplementation (20 mg/kg/day) prevented fructose-induced BP increase and reduced plasma cholesterol, high-density lipoprotein, and low-density lipoprotein. EC also improved NOS activity and eNOS phosphorylation and reduced superoxide production in the heart [73]. In addition, EC prevented NF-κB activation, reduced the overexpression of NOX, and enhanced SOD activity in the renal cortex [188]. Finally, in aged 18-month-old rats, EC (1 mg/kg/day for 15 days) improved blood NO levels and decreased hypertension [58].

Interestingly, TX (100 μg/kg/day) also reduced ACE activity in the aorta in young rats treated with dexamethasone at a dose of 30 μg/kg/day for 8 days. In 44-week-old rats, the 2-week intake of TX (100 μg/kg/day) decreased the ACE activity in the aortas to the level found in the young rats [187].

Collectively, these studies show the beneficial effects of EC and TX in various experimental models in which antioxidant effects and/or elevation of eNOS-derived NO release, mainly in the vasculature, account for the most frequently observed. These mechanisms increased NO bioavailability, which is a plausible explanation for the improvement in endothelial function and BP decrease. However, there are other health-promoting effects of EC and TX that participate in BP reduction and provide overall health benefits, as described above.

## 7. Conclusions

Various experimental studies have shown that EC and TX modify multiple cellular and molecular mechanisms that are significant in terms of the prevention or treatment and hypertension. These were mainly related to the antioxidant action of these flavonoids, the increase in endothelial NO production, and the reduction of ACE activity and iNOS expression. In addition, EC and TX possess anti-inflammatory, anticoagulant, and antithrombotic properties. These protective mechanisms of EC and TX contribute to the protection of endothelial function, and, thus, they can participate in the reduction of BP in various models of experimental hypertension independently of pathomechanism(s) involved in BP increase. In addition, it is important to note that both EC and TX do not produce significant toxic effects, even when administered at higher doses. The advantage of EC is that there is currently a large amount of data available on its antihypertensive and cardioprotective effects and the molecular mechanisms underlying them in various experimental models of hypertension. Data on EC antiviral effects are also available but not in relation to SARS-CoV-2. Nevertheless, the currently known multiple beneficial effects of EC can significantly contribute to the reduction of COVID-19 symptoms and may have a potential life-saving effect.

On the other hand, (−)-TX and (+)-TX were recognized as a possible inhibitors of SARS-CoV-2 replication in computer-based studies, which suggested TX as a natural substance effective for COVID-19 treatment, despite data suggesting that (+)-TX would be less effective. However, there are considerably fewer studies on the cardiovascular effects of TX in experimental models of hypertension, and the available studies mainly use (+)-TX. Thus, in the case of TX, the use of the racemic mixture (±)-TX could have a dual effect during SARS-CoV-2 infection: inhibition of the virus replication and improvement in cardiovascular functions, both of which may participate in improving the clinical condition and/or acceleration of COVID-19 treatment.

However, to date, there remains a lack of experimental in vivo studies supporting the predicted effects of TX in inhibition of SARS-CoV-2. In future studies, identification of the actions of TX and/or EC, in a suitable experimental model of COVID-19 with conditions of both normotension and hypertension, is recommended. Based on the current knowledge from preclinical studies, both TX and EC show strong potential for at least supportive care in the treatment of hypertension per se, but they may provide additional anti-inflammatory, antithrombotic and, in case of TX, also antiviral benefits that could be relevant to the treatment of hypertensive subjects suffering from SARS-CoV-2 infection.

## Figures and Tables

**Table 1:** Examples of the biological effects of (−)-epicatechin as well as (+)-taxifolin and/or (−)-taxifolin that were found in the experimental models and that may be relevant for the treatment of hypertension and viral infection.

Biological Effects	(−)-Epicatechin	(+)-Taxifolin and/or (−)-Taxifolin
Antihypertensive	[38,58,59,60,61,62,63,64]	[65,66]
Vascular	[38,58,59,67,68,69,70,71]	[66]
Cardioprotective	[72,73,74]	[75,76]
Antidiabetic, antihyperglycemic, or antidyslipidemic	[77,78,79,80,81]	[82]
Hepatoprotective	[79]	[83]
Respiratory	[84]	[85]
Antiinflammatory	[86,87,88]	[89,90]
Improved erythrocyte deformability	[38]	[91]
Antiaggregatory, antithrombotic, or anticoagulant	[38,92,93]	[64,94,95,96]
Antifibrotic	[84]	[75,97]
Antiviral	[13,14]	[17]

**Table 2:** Summary of cellular or molecular mechanisms underlying the biological effects of (−)-epicatechin as well as (+)-taxifolin and/or (−)-taxifolin.

Cellular or Molecular Mechanisms	(−)-Epicatechin	(+)-Taxifolin and/or (−)-Taxifolin
Chelation of Fe^2+^, Cu^2+^, Zn^2+^	[107]	[108]
ROS scavenging	[105,106]	[108]
Alterations of iron metabolism	[60,72]	[40,83]
Activation of antioxidant enzymes (SOD, CAT, or GPx)	[181,182]	[76,183]
Reduction of NOX or XO	[61,62,88,109,182,184]	[96,109,110]
Elevation of endothelial NO	[38,61,62,68,181]	[66]
Reduction of inducible NOS-produced NO	[87,90,131]	[90,129,130,131,185]
Arginase inhibition	[58,132,133]	[133]
Reduction of ACE	[160,161]	[65,186,187]
Nrf2 induction	[61,121]	[76,121,122]
NFκB reduction	[87,137,188]	[140]
Inflammasome reduction		[141,142]

Abbreviations: ACE, angiotensin-converting enzyme; CAT, catalase; GPx, glutathione peroxidase; NF-κB, nuclear factor-κB; NO, nitric oxide; NOS, nitric oxide synthase; NOX, nicotinamide adenine dinucleotide phosphate oxidase; Nrf2, nuclear factor erythroid-2 related factor 2; ROS, reactive oxygen species; SOD, superoxiddismutase; XO, xanthine oxidase.

## Abbreviations

ACE	angiotensin-converting enzyme
Ang II	angiotensin II
ARE	antioxidant response element
ATR	angiotensin receptor
BHRs	borderline hypertensive rats
CAT	catalase
COVID-19	coronavirus disease
COX	cyclooxygenase
CVDs	cardiovascular diseases
DOCA	deoxycorticosterone acetate
EC	(−)-epicatechin
EDCFs	endothelium-derived contracting factors
eNOS	endothelial NO synthase activity
GPx	glutathione peroxidase
HO-1	hemoxygenase-1
HUVEC	human umbilical veins endothelial cells
IL	interleukin
IUPAC	International Union of Pure and Applied Chemistry
Keap1	Kelch-like ECH-associating protein 1
L-NAME	NG-nitro-L-arginine methyl ester
MAPK	mitogen-activated protein kinase
M^pro^	main protease
NAD(P)H	nicotinamide adenine dinucleotide phosphate
NEP	neutral endopeptidase
NF-κB	nuclear factor-κB
NLRP3	nucleotide-binding oligomerization domain-, leucine-rich repeat- and pyrin domain-containing protein 3
NLRC4	neuronal apoptosis inhibitory protein (NAIP)/NLR family caspase activation and recruitment domain–containing protein 4
NO	nitric oxide
NODH	NO-deficient hypertension
eNOS	endothelial nitric oxide synthase
iNOS	inducible nitric oxide synthase
NOS	nitric oxide synthase
NOX	nicotinamide adenine dinucleotide phosphate oxidase
Nrf2	nuclear factor erythroid-2 related factor 2
PI3K/Akt/PKA	phosphatidylinositol-3-kinase/protein kinase B/protein kinase A
RAS	renin-angiotensin system
ROS	reactive oxygen species
SARS-CoV-2	severe acute respiratory syndrome corona virus 2
SHRs	spontaneously hypertensive rats
SOD	superoxide dismutase
SNS	sympathetic nervous system
TNFα	tumor necrosis factor α
TX	(+)-taxifolin, (−)-taxifolin or their racemic mixture
XO	xanthine oxidase
